# Human amnion-derived mesenchymal stem cells promote osteogenic differentiation of lipopolysaccharide-induced human bone marrow mesenchymal stem cells via ANRIL/miR-125a/APC axis

**DOI:** 10.1186/s13287-020-02105-8

**Published:** 2021-01-07

**Authors:** Yuli Wang, Fengyi Lv, Lintong Huang, Hengwei Zhang, Bing Li, Weina Zhou, Xuan Li, Yifei Du, Yongchu Pan, Ruixia Wang

**Affiliations:** 1 Jiangsu Key Laboratory of Oral Diseases, Nanjing Medical University, Nanjing, China; 2 Department of Oral and Maxillofacial Surgery, Affiliated Hospital of Stomatology, Nanjing Medical University, Nanjing, China; 3 Department of Pathology and Laboratory Medicine and Center for Musculoskeletal Research, University of Rochester Medical Center, Rochester, New York, USA; 4 Department of Temporomandibular Joint, Affiliated Hospital of Stomatology, Nanjing Medical University, Nanjing, China; 5 Department of Orthodontics, Affiliated Hospital of Stomatology, Nanjing Medical University, Nanjing, China; 6 Department of Dental Implant, Affiliated Hospital of Stomatology, Nanjing Medical University, Nanjing, China

**Keywords:** Adenomatous polyposis coli, β-Catenin, Human amnion-derived mesenchymal stem cells, Human bone marrow mesenchymal stem cells, Lipopolysaccharide, Long noncoding RNA ANRIL, microRNA-125a, Osteogenic differentiation

## Abstract

**Background and aim:**

Periodontitis is a chronic inflammatory disease inducing the absorption of alveolar bone and leading to tooth loss. Human amnion-derived mesenchymal stem cells (HAMSCs) have been used for studying inflammatory processes. This study aimed to explore the role of long noncoding RNA (lncRNA) antisense noncoding RNA in the INK4 locus (ANRIL) in HAMSC-driven osteogenesis in lipopolysaccharide (LPS)-induced human bone marrow mesenchymal stem cells (HBMSCs).

**Methods:**

The cells were incubated with a co-culture system. Reactive oxygen species (ROS) level and superoxide dismutase (SOD) activity were used to detect the oxidative stress level. Flow cytometry was performed to determine cell proliferation. The alkaline phosphatase (ALP) activity, Alizarin red assay, cell transfection, and rat mandibular defect model were used to evaluate the osteogenic differentiation. Quantitative real-time reverse transcription–polymerase chain reaction (RT-PCR), Western blot analysis, dual-luciferase reporter assay, and immunofluorescence staining were used to evaluate the molecular mechanisms.

**Results:**

This study showed that HAMSCs promoted the osteogenesis of LPS-induced HBMSCs, while the ANRIL level in HBMSCs decreased during co-culture. ANRIL had no significant influence on the proliferation of LPS-induced HBMSCs. However, its overexpression inhibited the HAMSC-driven osteogenesis in vivo and in vitro, whereas its knockdown reversed these effects. Mechanistically, this study found that downregulating ANRIL led to the overexpression of microRNA-125a (miR-125a), and further contributed to the competitive binding of miR-125a and adenomatous polyposis coli (APC), thus significantly activating the Wnt/β-catenin pathway.

**Conclusion:**

The study indicated that HAMSCs promoted the osteogenic differentiation of LPS-induced HBMSCs via the ANRIL/miR-125a/APC axis, and offered a novel approach for periodontitis therapy.

## Introduction

Periodontitis is a group of plaque-induced chronic inflammatory processes characterized by alveolar bone deficiency and tooth loss. So far, strategies for healing periodontitis are challenging; they may have remarkable individual differences and increase patients’ burden. Lipopolysaccharide (LPS), the major virulence factor of the outer membranes of Gram-negative bacteria, is related to the immune responses of periodontitis [1, 2]. Human bone marrow mesenchymal stem cells (HBMSCs) are commonly used in alveolar bone regeneration due to their self-renewal capacity and availability [3]. However, the LPS-induced pathological status is detrimental to HBMSC retention and survival. Human amnion-derived mesenchymal stem cells (HAMSCs), harvested in a noninvasive manner, have superior immunomodulatory properties and fewer ethical concerns [4]. A previous report showed that HAMSCs modulated the osteogenic differentiation and alleviated oxidative stress in LPS-induced HBMSCs [5]. Many studies explored the osteogenesis of HBMSCs against LPS by HAMSCs; however, the curative effect and regulatory mechanism still need to be fully investigated.

Long noncoding RNAs (lncRNAs) is a critical subgroup of noncoding RNAs (ncRNAs) more than 200 nucleotides in length [6]; they are involved in a broad spectrum of biological control and pathology [7, 8]. Antisense noncoding RNA in the INK4 locus (ANRIL) is an lncRNA first found in melanoma and neural system tumor [9]. Recent studies indicated the relationship between ANRIL and periodontitis [10, 11]. Higher level of ANRIL was found in *Porphyromonas gingivalis*-induced periodontal tissue [12]. This study investigated whether HAMSCs promoted the osteogenesis of LPS-induced HBMSCs via ANRIL.

MicroRNAs (miRNAs) are a class of small noncoding RNAs that activate the expression of protein-encoding genes by binding to the 3′-UTR of the target mRNAs [13]. Recent studies demonstrated that ANRIL participated in the malignant transformation and progression of various diseases by acting as a sponge of microRNA-125a (miR-125a) [14, 15]. However, whether the ANRIL/miR-125a axis is involved in the HAMSC-driven osteogenesis remains undetermined. This study investigated the effect of ANRIL and its underlying mechanism. HAMSCs promoted osteogenic differentiation in LPS-induced HBMSCs, while ANRIL had the opposite effect. Moreover, downregulating ANRIL induced miR-125a overexpression, further inhibiting adenomatous polyposis coli (APC) and activating Wnt/β-catenin signaling. These new references for the lncRNA–miRNA–mRNA functional network may be a powerful strategy for the therapy of periodontitis.

## Materials and methods

### Cell culture and co-culture system

All experiments were approved by the ethics and research committee of Nanjing Medical University (Permit Number: 2018-190). Informed consent was obtained from all the participants. HAMSCs were obtained from the discarded amniotic membrane, and HBMSCs were collected from patients undergoing sagittal split ramus osteotomy (SSRO). The cells were isolated and maintained as reported [16, 17]. The cells in three to five passages were used in this study. An HAMSCs/HBMSCs Transwell co-culture system was established as previously described [5]. HBMSCs were seeded at an initial cell density of 5 × 10^4^ cells/cm^2^ in six-well culture plates. Transwells were placed in other six-well culture plates, and HAMSCs were seeded at the same ratios (5 × 10^4^ cells/Transwell). Following the attachment of the cells (approximately 24 h), HBMSCs were subjected to a 24-h treatment with serum-free medium or *Escherichia coli* LPS (1 μg/mL) to induce inflammatory responses. After washing with phosphate-buffered saline, Transwells containing HAMSCs were transferred to the corresponding wells of the six-well culture plate containing HBMSCs.

### Quantitative real-time reverse transcription–polymerase chain reaction (RT-PCR)

RNA isolation and cDNA transcription were performed using TRIzol reagent (Invitrogen, NY, USA) and reverse transcription kit (Applied Biosystems, CA, USA). RT-PCR was conducted as previously reported [18]. The primer sequences used are listed in Table 1. Human glyceraldehyde-3-phosphate dehydrogenase was used as a reference for estimating the levels of lncRNAs and mRNAs, whereas human U6 was used to normalize miRNAs. The fold changes in gene expression were determined by the 2^−ΔΔCt^ method.

**Table 1:** Primers used for quantitative real-time reverse transcription polymerase chain reaction

Genes	Sense primer(5′-3′)	Anti-sense primer(5′-3′)
ALP	AGAACCCAAAGGCTTCTTC	CTTGGCTTTTCCTTCATGGT
RUNX2	TCTTAGAACAAATTCTGCCCTTT	TGCTTTGGTCTTGAAATCACA
OCN	AGCAAAGGTGCAGCCTTTGT	GCGCCTGGGTCTCTTCACT
OSX	CCTCCTCAGCTCACCTTCTC	GTTGGGAGCCCAAATAGAAA
ANRIL	CCCTAGCTACATCCGTCACCTGA	CCACAGCTACATATGCGTTTACA
APC	AAAGTGAGCAGCTACCACG	CCTGGAGTGATCTGTTAGTCG
훽-Catenin	AGCTGACAACTTTCACACC	AATGGGGATGTTGATCTTC

### Reactive oxygen species (ROS) level and superoxide dismutase (SOD) activity

Flow cytometry was used to determine LPS-induced ROS by measuring the intensity of 2′,7′-dichlorofluorescin fluorescence after 48 h as previously reported [5]. The SOD activity was detected using a xanthine oxidase assay kit (Jiancheng Corp., Nanjing, China) following the manufacturer’s protocols [19].

### Cell transfection

Recombinant lentiviruses containing full-length ANRIL, scramble control (NC), targeting ANRIL, and scramble control (shNC) were obtained from GenePharma Company (Shanghai, China). Those lentiviruses were named Lenti-ANRIL, Lenti-NC, Lenti-sh ANRIL, and Lenti-shNC, respectively. HBMSCs transfected with miRNA plasmids (RiboBio, Guangzhou, China) were prepared using transfection reagent riboFECTTM CP (RiboBio). The mutated binding sites of miR-125a in luciferase reporter vectors containing APC were constructed by site-directed mutagenesis.

### Cell proliferation assay

HBMSCs were collected after 1, 3, and 5 days. Flow cytometry (BD Biosciences, NJ, USA) was performed to determine the cell viability as previously reported [20]. G0, G1, S, and G2 M phases were determined using MODFIT LT 3.2 (Verity Software House, ME, USA).

### Alkaline phosphatase (ALP) and alizarin red assay

After 7 days of osteogenic induction, ALP staining was used to detect the activity with an NBT/BCIP staining kit (CoWin Biotech, Beijing, China) and an ALP assay kit (Jiancheng Corp, Nanjing, China) as previously reported [21, 22]. Mineralized matrix formation was determined after 14 days of osteogenic induction as previously reported [23].

### Western blot analysis

Western blot analysis was performed as previously reported [24]. The primary antibodies were as follows: anti-ALP (ab83259) (1:1000), anti-osteocalcin (OCN) (ab133612) (1:1000), and anti-Osterix (OSX) (ab209484) (1:1000) (all from Abcam, MA, USA; and RUNX2 (D1L7F) rabbit mAb #12556 (1:1000), APC antibody #2504 (1:1000), 훽-catenin (D10A8) XP Rabbit mAb #8480 (1:1000), and 훽-actin (8H10D10) mouse mAb #3700 (1:1000) (all from Cell Signaling Technology MA, USA). 훽-Actin served as an internal control. Relative densitometry analysis of the Western blot was carried out using ImageJ software. Relative protein levels were quantified as the ratio of the level of target protein to the level of 훽-actin, in each group.

### In vivo bone formation assay

Approximately 10^5^ cells (5 × 10^4^ HAMSCs and 5 × 10^4^ HBMSCs^NC^/HBMSCs^ANRIL^/HBMSCs^shNC^/HBMSCs^shANRIL^ pretreated with LPS) were attached to each HA/TCP biomaterial (Φ 5 × H 2 mm, Sichuan University, Chengdu, Sichuan, China). After 12 h, the complexes were subcutaneously implanted into the rat mandibular defect area designed as previously reported (four female nude rats per group, with an average weight of 280 g) [23]. All animal experiments were conducted in compliance with the regulations and guidelines of the Nanjing Medical University institutional animal care.

### Micro-computed tomography (micro-CT) analysis

Mandibles were harvested for micro-CT analysis after 8 weeks of implantation as previously reported [25]. Bone volume ratio (BV/TV, %) was calculated.

### Histological observation

Mandible samples were harvested and analyzed using hematoxylin and eosin (H&E), Masson trichrome, and immunohistochemical staining. Primary antibodies against RUNX2 (1:300 dilution) were used for immunohistochemical analysis as previously reported [23]. Positive areas were observed under the microscope.

### Dual-luciferase reporter assay

Luciferase assays were performed using the Lipofectamine 2000 and Dual-Luciferase Reporter Assay System as previously reported [26].

### Immunofluorescence staining

Primary antibody [훽-Catenin (D10A8) XP Rabbit mAb #8480 (1:100), Cell Signaling Technology], and DAPI were used to perform immunofluorescence staining as previously reported [23]. Images were captured under an inverted fluorescence microscope (Olympus, Japan).

### Statistical analysis

The data were expressed as the mean and standard deviation of at least three independent samples. Comparisons between two groups were analyzed using the two-tailed, unpaired Student’s *t* test. Comparisons among ≥ 3 groups were performed using one-way analysis of variance followed by Tukey’s multiple comparisons. *P* < 0.05 indicated a statistically significant difference.

## Results

### LncRNA-ANRIL expression in LPS-induced HBMSCs decreased with HAMSC co-culture

**Fig. 1:**
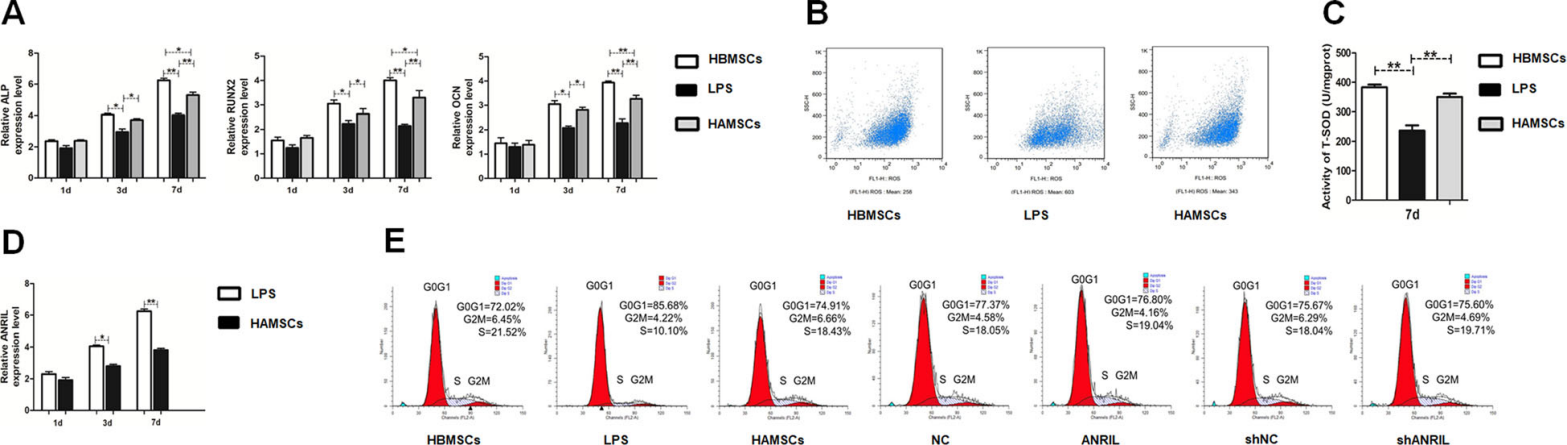
Osteogenic differentiation and oxidative stress level of HBMSCs co-cultured with HAMSCs, lncRNA-ANRIL expression in HBMSCs, and effects of lncRNA-ANRIL on the proliferation of HBMSCs. **a** Relative mRNA expression levels in HBMSCs were measured by RT-PCR analysis. **b**, **c** ROS and SOD levels in HBMSCs were measured using flow cytometry and xanthine oxidase assay kit. **d** LncRNA-ANRIL expression in HBMSCs was measured by RT-PCR analysis. **e** HBMSC proliferation was demonstrated by flow cytometry. Data are represented as mean ± standard error of the mean for all groups. ^*^P < 0.05 and ^**^P < 0.01. LPS: LPS + HBMSCs; HAMSCs, HAMSCs + LPS + HBMSCs; NC: HAMSCs + LPS + HBMSCs^NC^; ANRIL: HAMSCs + LPS + HBMSCs^ANRIL^; shNC: HAMSCs + LPS + HBMSCs^shNC^; shANRIL: HAMSCs + LPS + HBMSCs^shANRIL^

A Transwell co-culture model was established, and related markers were detected to verify the previous finding that HAMSCs reduced oxidative stress and promoted osteogenic differentiation in LPS-induced HBMSCs. HAMSCs increased the expression of osteogenic markers, including ALP, RUNX2, and OCN (Fig. 1a). In addition, HAMSCs also decreased the LPS-induced oxidative stress level in HBMSCs, which was confirmed by ROS and SOD measurements (Fig. 1b, c). Meanwhile, RT-PCR showed that the levels of ANRIL in LPS-induced HBMSCs significantly decreased in a time-dependent manner with the HAMSC co-culture (Fig. 1d).

### LncRNA-ANRIL had no effects on LPS-induced proliferation of HBMSCs

Then, stably expressing cells (HBMSCs^NC^, HBMSCs^ANRIL^, HBMSCs^shNC^, and HBMSCs^shANRIL^) were sorted and co-cultured with HAMSCs after LPS pretreatment. The cells were assigned to the following groups: HBMSCs; LPS: LPS + HBMSCs; HAMSCs: HAMSCs + LPS + HBMSCs; NC: HAMSCs + LPS + HBMSCs^NC^; ANRIL: HAMSCs + LPS + HBMSCs^ANRIL^; shNC: HAMSCs + LPS + HBMSCs^shNC^; shANRIL: HAMSCs + LPS + HBMSCs^shANRIL^. The proliferation assay conducted using flow cytometry suggested that HAMSCs promoted the growth of LPS-induced HBMSCs, while no significant difference in S-phase checkpoints was detected among the HAMSC, NC, ANRIL, shNC, and shANRIL groups (Fig. 1e).

### HAMSCs promoted the osteogenesis of LPS-induced HBMSCs via downregulating LncRNA-ANRIL

**Fig. 2:**
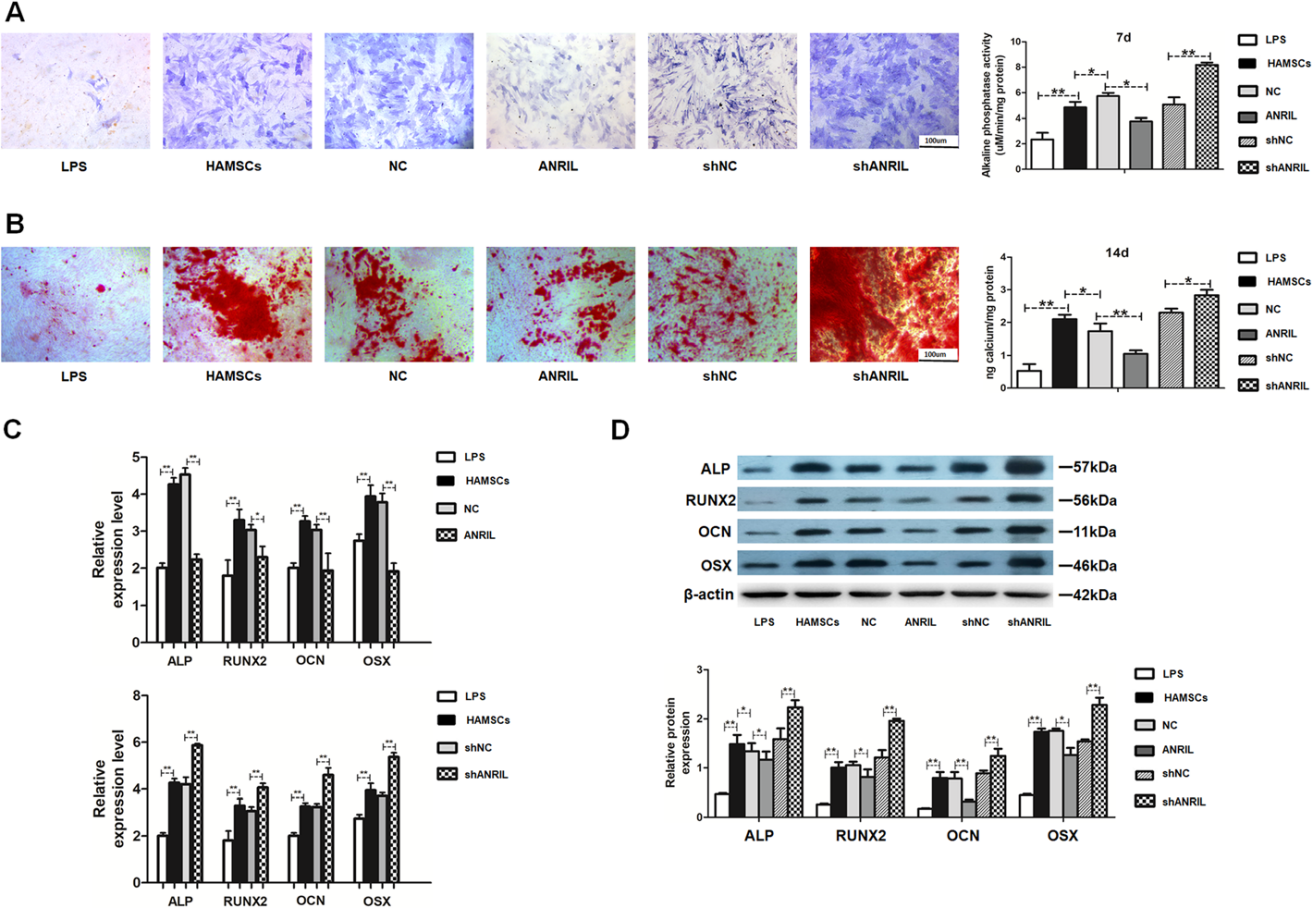
HAMSCs promoted the osteogenesis of LPS-induced HBMSCs via the downregulation LncRNA-ANRIL. **a** ALP staining and activity in HBMSCs. Scale bar, 100 μm. **b** Alizarin red staining and quantification in HBMSCs. Scale bar, 100 μm. **c** Relative mRNA expression levels in HBMSCs were measured by RT-PCR analysis. **d** Relative protein levels in HBMSCs were assessed by Western blot analysis. Data are represented as mean ± standard error of mean for all groups. ^*^P < 0.05 and ^**^P < 0.01. LPS: LPS + HBMSCs; HAMSCs: HAMSCs + LPS + HBMSCs; NC: HAMSCs + LPS + HBMSCs^NC^; ANRIL: HAMSCs + LPS + HBMSCs^ANRIL^; shNC: HAMSCs + LPS + HBMSCs^shNC^; shANRIL: HAMSCs + LPS + HBMSCs^shANRIL^

The osteogenic differentiation in HBMSCs is vital in reversing alveolar bone deficiency. Therefore, the present study investigated whether ANRIL participated in HAMSC-driven osteogenesis. HAMSCs enhanced ALP staining and activity compared with those in LPS groups, whereas ANRIL overexpression in HBMSCs inhibited the effect, and ANRIL knockdown promoted it (Fig. 2a). Besides, Alizarin red staining and quantification showed decreased matrix mineralization in the ANRIL group compared with the HAMSCs and NC groups, whereas ANRIL knockdown exhibited the opposite effects (Fig. 2b). RT-PCR and Western blot analysis showed that the mRNA and protein levels of ALP and RUNX2 (early-stage osteogenic markers) and OCN and OSX (late-stage markers) were decreased by ANRIL overexpression and increased by ANRIL knockdown (Fig. 2c, d).

**Fig. 3:**
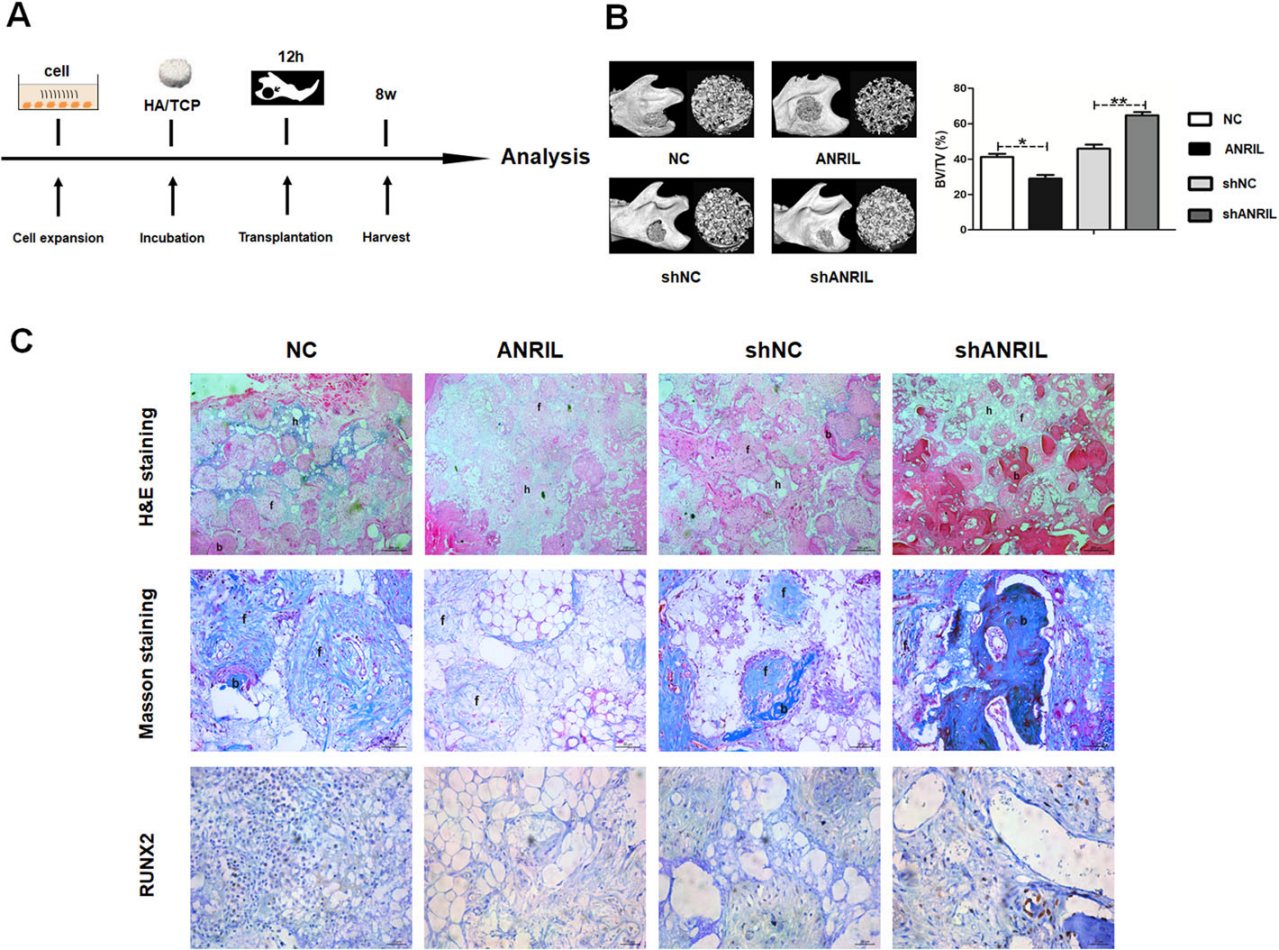
LncRNA-ANRIL in HBMSCs inhibited osteogenesis in vivo. **a** The cells in the NC, ANRIL, shNC, and shANRIL groups were transplanted subcutaneously into a rat critical-sized mandibular defect model. **b** Reconstructed 3D micro-CT images of the tissue-engineered bone and values of BV/TV. **c** H&E staining, Masson staining, and immunohistochemical staining of RUNX2 in each group (b: bone-like tissues; h: HA/TCP scaffold; f: fibrous). Scale bar, 200 μm. Data are represented as mean ± standard error of the mean for both groups. ^*^P < 0.05 and ^**^P < 0.01. NC: HAMSCs + LPS + HBMSCs^NC^; ANRIL: HAMSCs + LPS + HBMSCs^ANRIL^; shNC: HAMSCs + LPS + HBMSCs^shNC^; shANRIL: HAMSCs + LPS + HBMSCs^shANRIL^

Followed the in vitro study, this study examined the effect of ANRIL in vivo using the mandibular defect model (Fig. 3a). The percentage of mineralized volume fraction was measured. ANRIL decreased bone volume/total volume (BV/TV) compared with the NC group, while increased BV/TV was detected in the shANRIL group compared with shNC group (Fig. 3b). H&E and Masson staining showed less organized bone matrix and more fibrous tissue in the ANRIL group, while the shANRIL group exhibited better bone formation. The immunohistochemical analysis also confirmed that RUNX2 expression was downregulated in the ANRIL group compared with the NC group and upregulated in the shANRIL group compared with the shNC group (Fig. 3c).

### MiR-125a negatively correlated with LncRNA-ANRIL

**Fig. 4:**
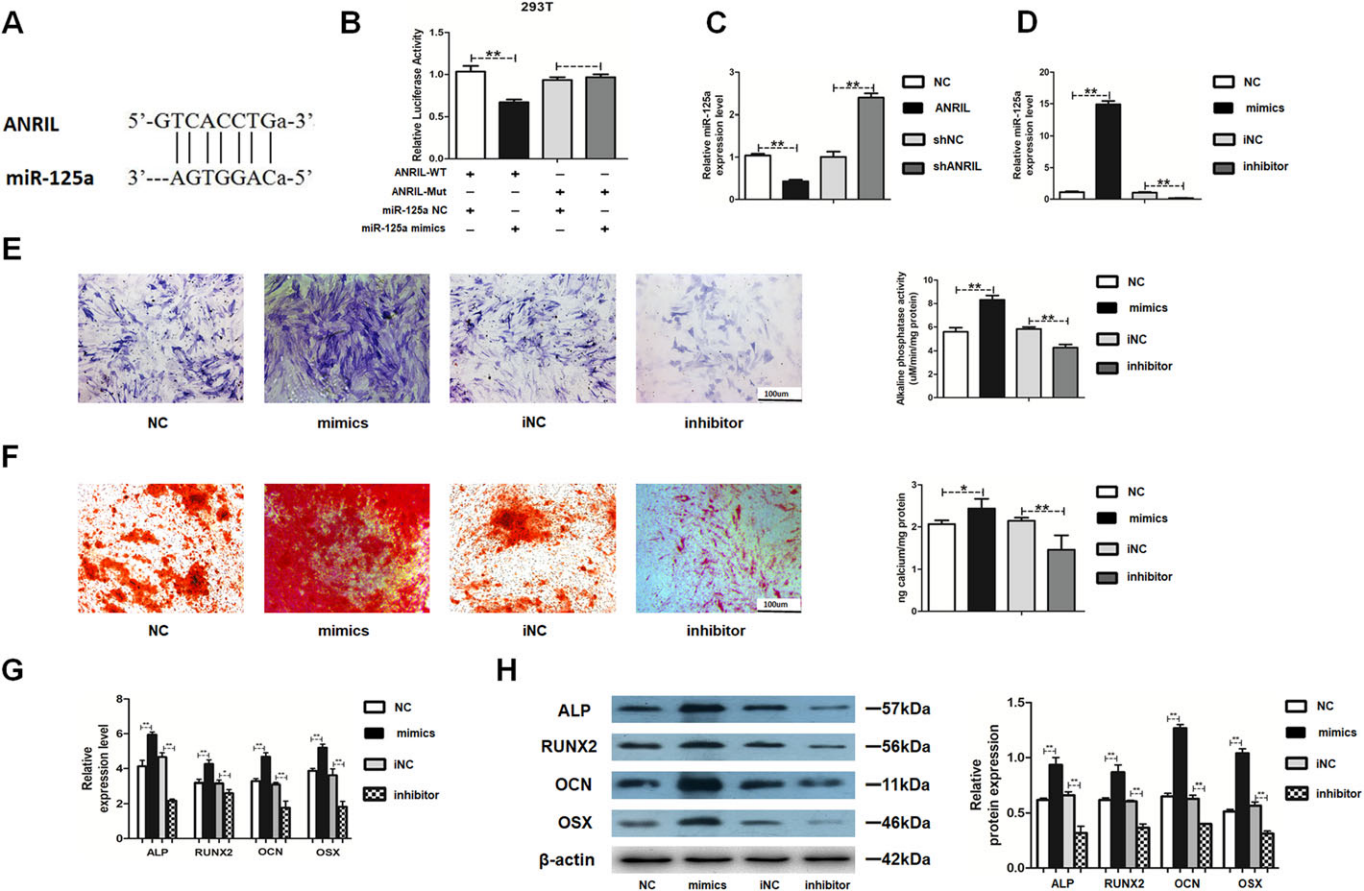
MiR-125a in HBMSCs was regulated by lncRNA-ANRIL and promoted the osteogenic differentiation of HBMSCs. **a** The binding sequence between miR-125a and ANRIL predicted using biological software. **b** Luciferase reporter assay was used to verify the correlation between ANRIL and miR-125a in 293T cells. **c** MiR-125a expression was measured by RT-PCR in the NC, ANRIL, shNC, and shANRIL groups. NC: HAMSCs + LPS + HBMSCs^NC^; ANRIL: HAMSCs + LPS + HBMSCs^ANRIL^; shNC: HAMSCs + LPS + HBMSCs^shNC^; shANRIL: HAMSCs + LPS + HBMSCs^shANRIL^. **d** Transfection efficacy of miR-125a was detected by RT-PCR. **e** ALP staining and activity in the NC, mimics, iNC, and inhibitor groups. Scale bar, 100 μm. **f** Alizarin red staining and quantification in the NC, mimics, iNC, and inhibitor groups. Scale bar, 100 μm. **g** Relative mRNA expression levels were measured by RT-PCR analysis. **h** Relative protein levels were assessed by Western blot analysis. Data are represented as mean ± standard error of the mean for both groups. ^*^P < 0.05 and ^**^P < 0.01. NC: HAMSCs + LPS + HBMSCs^miR-125a NC^; mimics: HAMSCs + LPS + HBMSCs^miR-125a mimics^; iNC: HAMSCs + LPS + HBMSCs^miR-125a iNC^; inhibitor: HAMSCs + LPS + HBMSCs^miR-125a inhibitor^

This study next investigated the possible interaction between ANRIL and miR-125a. The putative miR-125a-binding sequence of ANRIL was predicated using TargetScan software (Fig. 4a). The correlation between ANRIL and miR-125a was further examined using a dual-luciferase reporter assay to identify whether ANRIL could serve as an miRNA sponge to directly negatively regulate the expression of miR-125a. The results showed that miR-125a significantly attenuated the luciferase activity of ANRIL-WT, while no change was detected in ANRIL-Mut groups (Fig. 4b). The miR-125a expression was downregulated by ANRIL overexpression and upregulated by ANRIL knockdown (Fig. 4c). Further, miR-125a mimics and inhibitor were transfected into HBMSCs, and the transfection efficacy was detected by RT-PCR (Fig. 4d). The cells were assigned to the following groups: NC: HAMSCs + LPS + HBMSCs^miR-125a NC^; mimics: HAMSCs + LPS + HBMSCs^miR-125a mimics^; iNC: HAMSCs + LPS + HBMSCs^miR-125a iNC^; inhibitor: HAMSCs + LPS + HBMSCs^miR-125a inhibitor^. Moreover, miR-125a mimics enhanced ALP staining and activity compared with those in the NC groups, whereas miR-125a inhibitor in HBMSCs inhibited the effect (Fig. 4e). Besides, Alizarin red staining and quantification showed increased matrix mineralization in the miR-125a mimics group compared with the NC groups, whereas miR-125a inhibitor showed the opposite effects (Fig. 4f). RT-PCR and Western blot analysis showed that the mRNA and protein levels of ALP and RUNX2 (early-stage osteogenic markers) and OCN and OSX (late-stage markers) were increased by miR-125a mimics and decreased by miR-125a inhibitor (Fig. 4g, h).

### MiR-125a alleviated HAMSC-driven osteogenesis in HBMSCs by targeting APC and activating the Wnt/β-catenin pathway

**Fig. 5:**
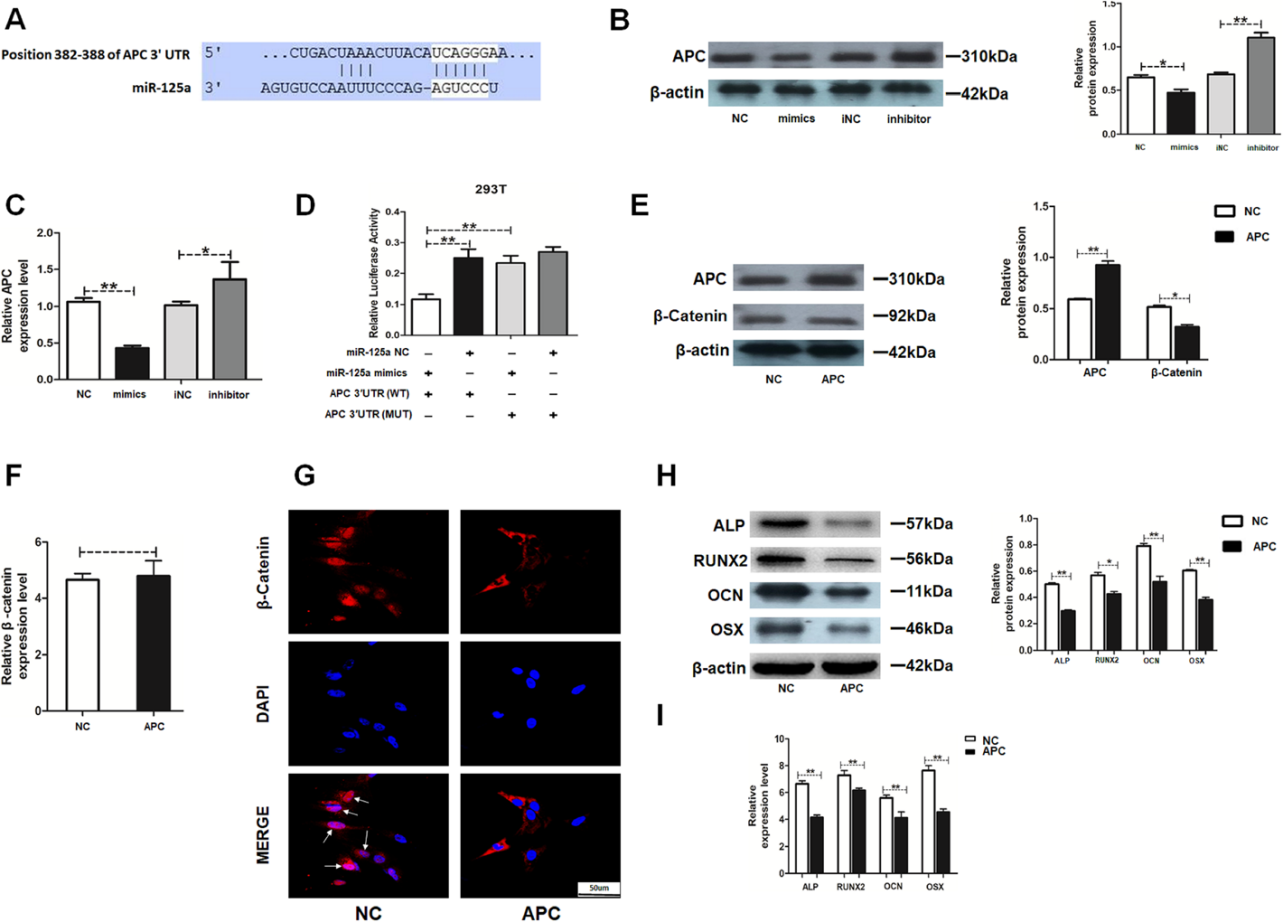
MiR-125a targeted APC, activated Wnt/β-catenin pathway, and alleviated osteogenesis. **a** The potential binding sites between APC and miR-125a predicted using biological software. **b** APC protein level was assessed by Western blot analysis in the NC, mimics, iNC, and inhibitor groups. **c** APC mRNA expression was measured by RT-PCR analysis in the NC, mimics, iNC, and inhibitor groups. NC: HAMSCs + LPS + HBMSCs^miR-125a NC^; mimics: HAMSCs + LPS + HBMSCs^miR-125a mimics^; iNC: HAMSCs + LPS + HBMSCs^miR-125a iNC^; inhibitor: HAMSCs + LPS + HBMSCs^miR-125a inhibitor^. **d** Luciferase reporter assay was used to validate the target in 293T cells. Relative Renilla luciferase activity was normalized to that of firefly luciferase. **e** APC protein level was assessed by Western blot analysis in the NC and APC groups. **f** Relative mRNA expression of β-catenin was measured by RT-PCR analysis in the NC and APC groups. **g** Immunofluorescence staining showed a β-catenin location in the NC and APC groups. Scale bar, 50 μm. **h** Relative protein levels were assessed by Western blot analysis in the NC and APC groups. **i** Relative mRNA expression levels were measured by RT-PCR analysis in the NC and APC groups. NC: HAMSCs + LPS + HBMSCs^APC NC^; APC: HAMSCs + LPS + HBMSCs^APC^. Data are represented as mean ± standard error of the mean for both groups. ^*^P < 0.05 and ^**^P < 0.01

APC was selected as the candidate target gene of miR-125a using TargetScan software (Fig. 5a). Western blot assay and RT-PCR showed that the mRNA and protein levels of APC were significantly decreased by miR-125a mimics and increased by the miR-125a inhibitor (Fig. 5b, c). The study further explored the mechanisms underlying miR-125a–APC interaction, and a dual-luciferase reporter assay was performed. The results suggested that miR-125a mimics reduced the luciferase activity of APC wild-type reporter, while this suppressive effect was rescued by mutation of the putative miR-125a target sites (Fig. 5d).

As APC is a negative regulator in the Wnt/β-catenin pathway [27], APC overexpression was established in HBMSCs and the cells were assigned to the following groups: NC: HAMSCs + LPS + HBMSCs^APC NC^; APC: HAMSCs + LPS + HBMSCs^APC^. Western blot analysis showed that APC overexpression increased the protein level of APC and decreased the protein level of β-catenin (Fig. 5e). However, the mRNA level of β-catenin was stable, suggesting that APC overexpression induced only β-catenin protein degradation without influencing mRNA (Fig. 5f). Immunofluorescence staining further showed a significant decrease in nuclear β-catenin accumulation when APC was overexpressed (Fig. 5g). Moreover, Western blot analysis and RT-PCR showed that the protein and mRNA levels of ALP and RUNX2 (early-stage osteogenic markers) and OCN and OSX (late-stage markers) were decreased by APC overexpression (Fig. 5h, i).

### MiR-125a inhibitor suppressed the APC downregulation and osteogenesis caused by shANRIL

**Fig. 6:**
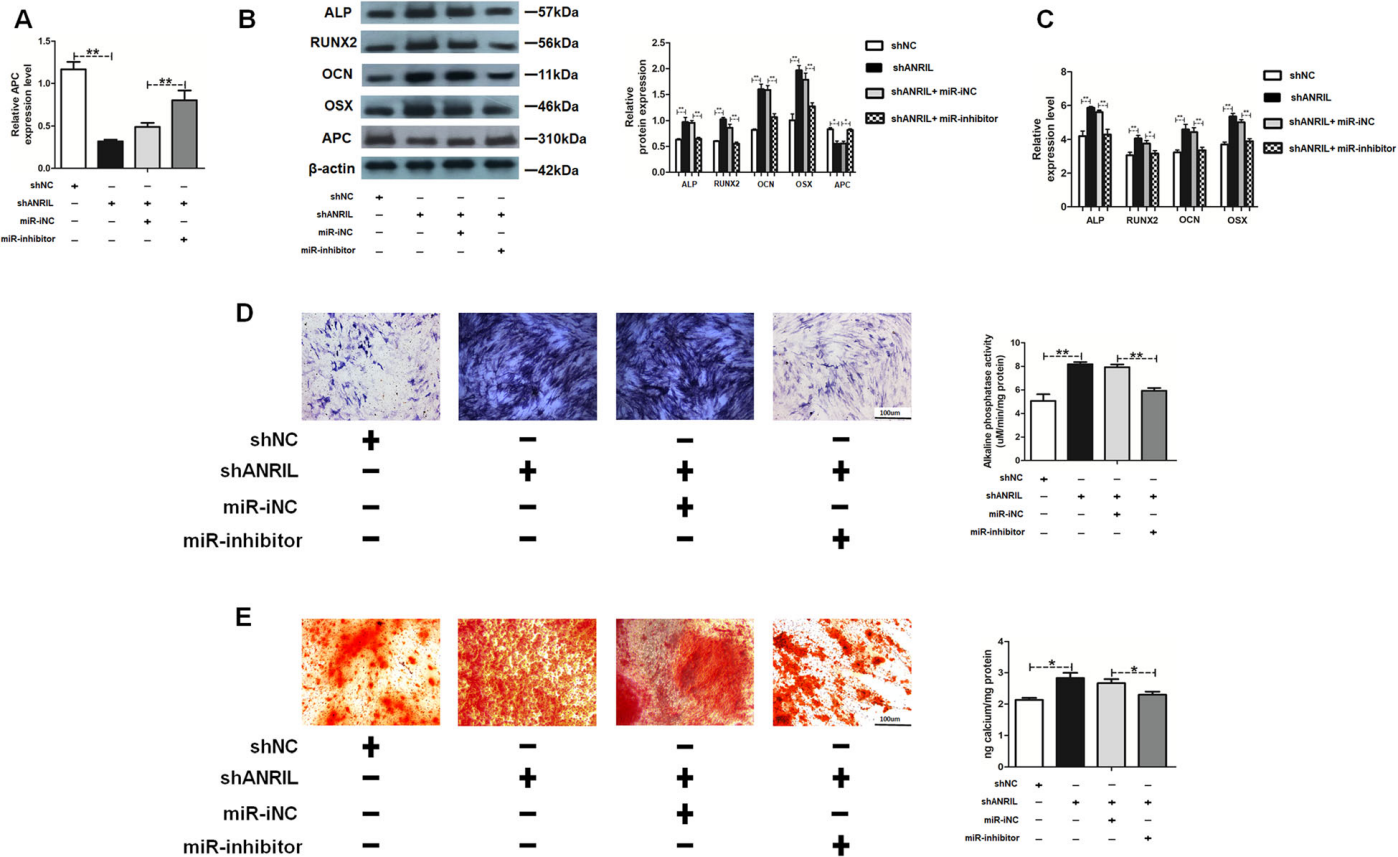
MiR-125a inhibitor could suppress the shANRIL-mediated positive effects. **a** APC mRNA expression was measured by RT-PCR analysis. **b** Protein levels were assessed by Western blot analysis. **c** Relative mRNA expression levels were measured by RT-PCR analysis. **d** ALP staining and activity in each group. Scale bar, 100 μm. **e** Alizarin red staining and quantification in each group. Scale bar, 100 μm. Data are represented as mean ± standard error of the mean for both groups. ^*^P < 0.05 and ^**^P < 0.01

The rescue assays were carried out to fully understand the role of miR-125a and APC in ANRIL-mediated osteogenesis. As shown in Fig. 6a, b, shANRIL and miR-125a inhibitor co-transfection rectified APC suppression compared with that in the shANRIL group. The shANRIL-mediated expression of osteogenic markers was also partly inhibited in co-transfected cells (Fig. 6b, c). shANRIL enhanced ALP staining and activity compared with those in the shNC groups, whereas co-transfection partly inhibited the effect (Fig. 6d). Besides, Alizarin red staining and quantification showed decreased matrix mineralization in the co-transfection group compared with those in the shANRIL groups (Fig. 6e). Taken together, HAMSCs downregulated the expression of lncRNA-ANRIL in LPS-induced HBMSCs, upregulated the miR-125a level, targeted APC transcription, and activated the Wnt/β-catenin pathway to induce new bone formation.

## Discussion

**Fig. 7:**
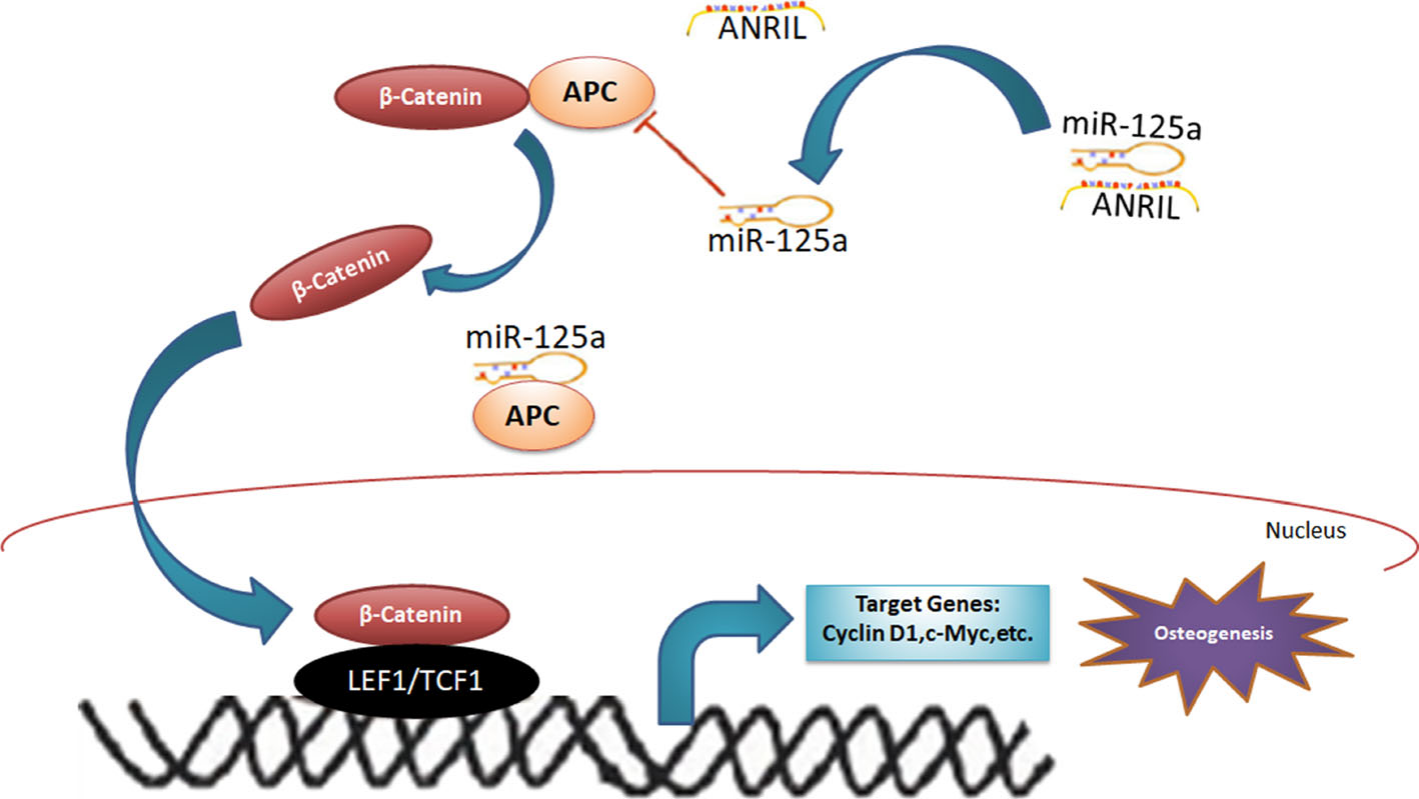
Schematic working model for lncRNA-ANRIL/miR-125a/APC/β-catenin axis in the regulation of LPS-induced HBMSC osteogenesis

The present study demonstrated that lncRNA-ANRIL derived from LPS-pretreated HBMSCs was responsible for HAMSC-induced osteogenesis. Briefly, HAMSCs promoted osteogenesis and reduced oxidative stress in LPS-pretreated HBMSCs, and the expression of lncRNA-ANRIL in HBMSCs significantly decreased with HAMSC co-culture. Then, the application of HAMSCs + LPS + HBMSCs^shANRIL^ into the scaffold material induced bone formation in mandibular defects. Furthermore, miR-125a, acting as a sponge of ANRIL, not only regulated HBMSC function in osteogenesis by targeting APC but also positively regulated osteogenic differentiation via the Wnt/β-catenin pathway, suggesting that the axis might be a potentially biological target for treating inflammation-related bone deficiency (Fig. 7). Periodontitis is the most prevalent disease of the alveolar bone, which eventually leads to periodontal tissue and tooth loss. Yet, the current strategies, such as autologous bone and biomaterial transplantation, suffer from limited sources, uncertain outcomes, and high cost [28, 29]. Tissue regeneration based on stem cells is a promising therapeutic approach. HAMSCs, isolated from the amniotic membrane, is associated with superior immunomodulatory properties and less ethical controversy [4, 30]. A previous study also indicated that decreased inflammation factors and increased growth factors were the potential causes of HAMSC implication in tissue remodeling [30]. At the beginning of the study, it was observed that the osteogenic differentiation and oxidative stress of LPS-induced HBMSCs were significantly alleviated with HAMSCs co-culture, which was consistent with a previous investigation [31]. Importantly, these phenotypic changes in LPS-induced HBMSCs were accompanied by progressively reduced lncRNA-ANRIL expression.

Next, lncRNA-ANRIL was explored in this study. ANRIL, which was formerly regarded as the risk locus associated with coronary heart disease and germline deletion by genome-wide association studies [9, 32], is located in a 42-kb stretch within the chromosomal region 9p21.3. Periodontitis and coronary heart disease share similar pathogenic bacterial strains and environmental and behavioral risk factors [33, 34]. Hence, a recent study suggested a positive correlation between ANRIL and periodontitis across different populations [11]. Furthermore, enhanced ANRIL transcription in bacteria-infected periodontal tissue was upregulated by multiple signaling pathways in response to inflammation [35]. Considering the anti-inflammatory properties of HAMSCs and decreased ANRIL expression in LPS-induced HBMSCs, the present study tested whether HAMSCs performed their function through ANRIL. In this study, HBMSCs^ANRIL^ led to reversed osteogenesis, while HBMSCs^shANRIL^ displayed the opposite effects. These data demonstrated that HAMSCs contributed to LPS-induced HBMSC osteoblastogenesis by reducing ANRIL expression.

In the last few years, lncRNAs have been indicated as miRNA sponges to negatively regulate miRNA expression [36]. MiRNAs are involved in multiple cellular processes by influencing gene expression [37]. Thus, it was hypothesized that the effects of ANRIL might be associated with miRNA expression. MiR-125a can participate in cell proliferation, differentiation, and senescence by influencing mRNA stability and translation [38]. The inhibition of ANRIL induces cell apoptosis, which may have a correlation with increased miR-125a expression [15]. Additionally, the association between miR-125a and periodontitis has been investigated in a previous study [39]. The present study demonstrated that miR-125a in LPS-induced HBMSCs was directly bound by ANRIL and negatively regulated,while ANRIL was not affected by miR-125a (see Supplementary Material). MiR-125a mimics increased osteogenesis, and miR-125a inhibitor reversed osteogenic differentiation caused by HAMSCs. Collectively, all these data indicated that ANRIL acted as an miR-125a sponge in the underlying mechanism.

The Wnt/β-catenin pathway is a classic signal having multiple roles in cellular behaviors [40, 41]. APC, which acts as a negative regulator in the Wnt/β-catenin pathway, notably binds to β-catenin and inhibits its transfer [42]. Targeting APC may emerge as external stimuli, promote the nuclear localization of β-catenin, and activate the pathway. The present study confirmed that miR-125a bound to the 3′-UTR of APC, and their expression negatively correlated. Then, the study explored the roles of APC and β-catenin in the underlying mechanism. The results suggested that APC overexpression intuitively decreased the nuclear localization of β-catenin and contributed to osteoblast repression in HBMSCs. Moreover, the rescue assays showed that shANRIL and miR-125a inhibitor co-transfection rectified APC suppression and inhibited osteogenic differentiation. Thus, it is believed that the interaction between APC and β-catenin serves as a downstream regulator of miR-125a in ANRIL-induced osteogenesis.

## Conclusions

In conclusion, the findings of the present study supported the hypothesis that HAMSCs promoted the osteogenic differentiation of LPS-induced HBMSCs via the lncRNA-ANRIL/miR-125a/APC/β-catenin axis. Based on these results, a viable therapeutic target and paramount approach may be proposed for the therapy of inflammatory bone-destructive processes.

## Supplementary Information

**Additional file 1.** Other relevant datasets

## Data Availability

All other relevant datasets have been uploaded as part of additional files.

## Abbreviations

ALP: Alkaline phosphatase
ANRIL: Antisense noncoding RNA in the INK4 locus
APC: Adenomatous polyposis coli
BV/TV: Bone volume/total volume
DCF: 2′,7′-Dichlorofluorescein
GAPDH: Glyceraldehyde-3-phosphate dehydrogenase
HAMSCs: Human amnion-derived mesenchymal stem cells
HBMSCs: Human bone marrow mesenchymal stem cells
H&E: Hematoxylin and eosin
lncRNAs: Long noncoding RNAs
LPS: Lipopolysaccharide
micro-CT: Micro-computed tomography
miR-125a: MicroRNA-125a
miRNAs: MicroRNAs
NcRNAs: Noncoding RNAs
OCN: Osteocalcin
OSX: Osterix
ROS: Reactive oxygen species
RT-PCR: Quantitative real-time reverse transcription–polymerase chain reaction
SOD: Superoxide dismutase
SSRO: Sagittal split ramus osteotomy
