# Supplementary figures and images for: Human amnion-derived mesenchymal stem cells promote osteogenic differentiation of lipopolysaccharide-induced human bone marrow mesenchymal stem cells via ANRIL/miR-125a/APC axis
Source: Stem Cell Res Ther. 2021 Jan 7;12:35. doi: 10.1186/s13287-020-02105-8 (PMC7791649; doi:10.1186/s13287-020-02105-8)

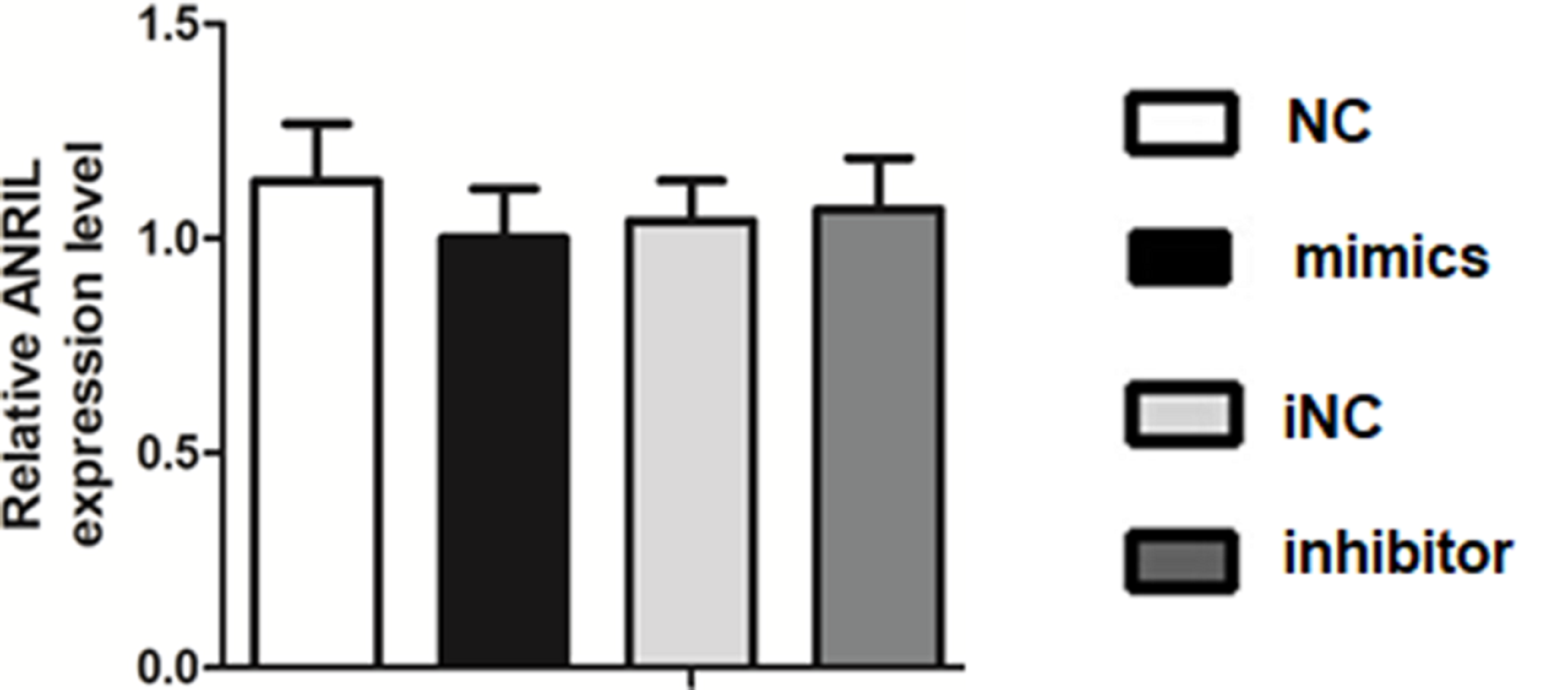

Supplement: Supplementary file 1 — Additional file 1. Other relevant datasets [file 13287_2020_2105_MOESM1_ESM.tif]
